# Combination of Dendrobium Mixture and Metformin Curbs the Development and Progression of Diabetic Cardiomyopathy by Targeting the lncRNA NEAT1

**DOI:** 10.6061/clinics/2021/e2669

**Published:** 2021-06-29

**Authors:** Wenmin Qin, Xing Zhao, Jie Tai, Guoyun Qin, Shanshan Yu

**Affiliations:** I Department of Pharmacy, The Second People's Hospital of Dongying, Dongying, Shandong 257335, China.; II Department of Pharmacy, The Fifth Central Hospital of Tianjin, Tianjin 300450, China.; III Department of Rehabilitation Medicine, The Second People's Hospital of Liaocheng, Liaocheng, Shandong 252000, China.; IV Department of Pharmacy, Yidu Central Hospital of Weifang, Qingzhou, Shandong 262500, China.

**Keywords:** Diabetic Cardiomyopathy, Dendrobium Mixture, Metformin, *NEAT1*, Nrf2, *miR-23a-3p*

## Abstract

**OBJECTIVES:**

This study aimed to explore the efficacy of combination treatment with dendrobium mixture and metformin (Met) in diabetic cardiomyopathy (DCM) and its effects on NEAT1 and the Nrf2 signaling pathway.

**METHODS:**

H9c2 cells were maintained in medium supplemented with either low (5.5 mmol/L) or high (50 mmol/L) glucose. Male Sprague-Dawley rats were fed a high-glucose diet and administered a single, low dose of streptozotocin (35 mg/kg) via intraperitoneal injection to induce the development of DM. After induction of DM, the rats were treated with dendrobium mixture (10 g/kg) and Met (0.18 g/kg) daily for 4 weeks. Next, quantitative reverse transcription (qRT)-PCR and western blotting were performed to evaluate the expression levels of target genes and proteins. Flow cytometry was performed to assess apoptosis, and hematoxylin and eosin staining was performed to evaluate the morphological changes in rat cardiac tissue.

**RESULTS:**

In patients with diabetes mellitus (DM) and myocardial cells and heart tissues from rats with high glucose-induced DM, *NEAT1* was downregulated, and the expression levels of Nrf2 were decreased (*p*<0.01, *p*<0.001). The combination of dendrobium mixture and Met upregulated the expression of *NEAT1* which upregulated Nrf2 by targeting *miR-23a-3p*, resulting in reduced apoptosis and improved cardiac tissue morphology (*p*<0.01, *p*<0.001).

**CONCLUSION:**

Dendrobium mixture and Met upregulated the expression of *NEAT1* in DCM, thereby inhibiting apoptosis of myocardial cells.

## INTRODUCTION

Diabetic cardiomyopathy (DCM) is the most common complication of diabetes mellitus (DM) and is a major cause of DM-related mortality. DCM, which is estimated to affect approximately 60% of patients with well-managed DM (1,2), presents with the characteristic features of ventricular dysfunction, myocardial interstitial fibrosis, and myocardial hypertrophy, and has been shown to be distinct from heart disease caused by coronary artery disease or hypertension (3,4).

Increasing evidence suggests that the hyperglycemia-induced generation of reactive oxygen species (ROS) is a cause of DCM progression (5-7). An acute increase in ROS induces the generation of multiple cytokines and inflammatory factors, including nuclear factor kappa B and thioredoxin-interacting protein/inhibitory protein (6,8). Oxidative stress can be reduced by the intracellular factor nuclear factor erythroid-derived 2-like 2 (Nrf2) (9,10). Thus, activation of Nrf2 is an efficient strategy for protecting myocardial cells against ROS-induced oxidative stress.

The long non-coding RNA (lncRNA) nuclear enriched transcription factor 1 (*NEAT1*) is an important component of paraspeckles, as it plays an indispensable role in their formation and maintenance. Studies have shown that *NEAT1* plays regulatory roles in various cancers, and high expression of *NEAT1* is strongly associated with poor prognosis. However, in leukemia, decreased *NEAT1* levels impaired the myeloid differentiation of acute promyelocytic leukemia cells (11).

Few studies have examined the role of *NEAT1* in DCM; thus, the regulatory mechanism of *NEAT1* in the occurrence and development of DCM is unknown. However, in a recent study, *NEAT1* expression was shown to be downregulated in a mouse model of type 1 (T1) DM with cardiac dysfunction (12), which suggests that *NEAT1* may play a positive regulatory role in DCM.

Dendrobium mixture is a traditional Chinese medicine that has been used for the treatment of DM. It consists of dendrobe, *Astragalus membranaceus*, and *Salvia miltiorrhiza* and has been shown to be effective for the treatment of DM-induced dryness-heat, polydipsia, soreness of the waist, tinnitus, deficiency-heat, and night sweats; it also has anti-oxidative effects (13,14). Metformin (Met) is a hypoglycemic drug that is commonly prescribed for the treatment of DM worldwide (15). In addition, a growing body of evidence has suggested the efficacy of Met for the treatment of other diseases, including endometrial and lung cancer. Met has also shown promise for protecting myocardial cells from damage (15-18).

In this study, we aimed to evaluate the efficacy of dendrobium mixture in combination with Met for ameliorating myocardial cell apoptosis and fibrosis in a rat model of DCM. We also assessed whether this treatment combination could alleviate DCM-induced injury by modulating the expression of Nrf2. Our study revealed that *NEAT1* was downregulated in DCM rats, and the combination of dendrobium and Met altered the levels of Nrf2 and *miR-23a-3p* by regulating *NEAT1* expression levels in myocardial cells from rats with high-glucose (HG) diet-induced DM and in rats with streptozotocin (STZ)-induced DCM. This was accompanied by a significant reduction in DCM-related symptoms. Thus, the results of this study demonstrated that the combination of dendrobium mixture and Met is a promising treatment candidate for DCM.

## MATERIALS AND METHODS

### Subjects and ethical statement

Serum samples were collected from eight patients with DM and eight healthy subjects admitted to the Second People’s Hospital of Dongying. The patients enrolled in this study had no coronary heart disease, hypertension, or other heart disease and were clinically diagnosed with typical symptoms of DM (polyuria, polydipsia, weight loss, and fasting blood glucose ≥8.0 mmol/L). The eight healthy subjects had no symptoms of DM and no family history of DM. All study subjects provided written informed consent, and the study protocol was approved by the Ethical Board of Second People’s Hospital of Dongying (approval no. KY2017-189).

### RNA extraction and quantitative reverse transcription (qRT)-PCR

Total RNA was extracted from serum samples and myocardial cells using TRIzol LS Reagent (Invitrogen, Carlsbad, CA, USA), and RNA purity was evaluated using a NanoDrop spectrometer (NanoDrop Technologies, Wilmington, DE, USA). The extracted RNA was reverse transcribed to cDNA using the reverse transcription kit (Toyobo, Japan). Next, the cDNA samples were amplified by qPCR using SYBR Green I (Yoyobo, Osaka, Japan) as the fluorescent base on an ABI 7500 PCR system, with the following cycling parameters: 95°C for 60s, followed by 40 cycles of 95°C for 15s, 40°C for 15s, and 72°C for 45s. The relative expression of each target gene was determined using the 2^-ΔΔCt^ method. The TaqMan microRNA cDNA synthesis kit (Biolab, Beijing, China) was used to measure *miR-23a-3p* expression levels. Target RNA expression levels were normalized to *U6* and *GAPDH* levels. The sequences of the PCR primers used were as follows:

*NEAT1* forward: 5′- TGGCTAGCTCAGGGCTTCAG-3′;

*NEAT1* reverse: 5′- TCTCCTTGCCAAGCTTCCTT-3′;

*HO-1* forward: 5′-TGAAGGAGGCCACCAAGGAGGA-3′;

*HO-1* reverse: 5′-AGAGGTCACCCAGGTAGCGGG-3′;

*MDA* forward: 5′-TCTTCACAAATCCTCCCC-3′;

*MDA* reverse: 5′-TGGATTAAAAGGACTTGG-3′;

*Nrf2* forward: 5′-CTGAACTCCTGGACGGGACTA-3′;

*Nrf2* reverse: 5′-CGGTGGGTCTCCGTAAATGG-3′;

*GAPDH* forward: 5′-CAGTGCCAGCCTCGTCTCAT-3′; and

*GAPDH* reverse: 5′-AGGGGCCATCCACAGTCTTC-3′.

### Western blotting

Proteins extracted from either tissue specimens or serum samples were loaded into the lanes (100 μg per lane) of a 10% SDS-polyacrylamide gel and separated by electrophoresis, and then transferred onto PVDF membranes. After the membranes were blocked with 5% non-fat milk for 2h, they were incubated with the primary antibodies at 4°C overnight, and then with the secondary antibodies at room temperature for 1h. The resulting immunoblots were developed with ECL reagent and imaged using an Odyssey Infrared Imaging System (Licor, USA). Antibodies against Nrf2, HO-1, MDA, and β-actin and corresponding secondary antibodies were purchased from Santa Cruz Biotechnology (Dallas, TX, USA).

### Preparation of dendrobium mixture

Dendrobium mixture, consisting of *Dendrobium officinale*, *Astragalus membranaceus*, *Salvia miltiorrhiza*, *Pueraria lobata*, *Schisandra chinensis*, *Rehmannia glutinosa*, *Cyathula officinalis*, *Rhizoma anemarrhenae*, earthworm, and *Oldenlandia diffusa*, was prepared using water extraction and alcohol precipitation methods. The crude drug concentration in the resulting preparation was 2.0 g/mL.

### Cell culture

H9c2 rat cardiomyocytes were obtained from the China Center for Type Culture Collection (Wuhan, China) and maintained in Dulbecco’s modified Eagle’s medium (Hyclone, Logan, UT, USA) supplemented with 10% fetal bovine serum (Gibco, Waltham, MA, USA), 1% penicillin (Gibco), and 1% streptomycin. For the experiments, the cells were transferred to the following experimental media: low-glucose medium (5.5 mmol/L, control group), high-glucose medium (50 mmol/L, HG group), high-glucose medium supplemented with Met (2.5 mmol/L; Meilunbio, Dalian, China; Met group), high-glucose medium supplemented with dendrobium mixture (2.0 g/mL, DN group), and high-glucose medium supplemented with dendrobium mixture and Met (treatment group).

### Flow cytometry and MTT assay

Cells from each group were resuspended in 100 μL of annexin binding buffer and mixed with 5 μL of propidium iodide (PI) and 5 μL of FITC-conjugated annexin V (BD Biosciences, Franklin Lakes, NJ, USA) and incubated for 20 min in the dark at room temperature. Next, annexin binding buffer (400 μL) was added, and the fluorescence signals for PI and annexin V were detected by flow cytometry. 3‐(4, 5‐Dimethylthiazol‐2‐yl)‐2, 5‐diphenyltetrazolium bromide (MTT) assays were performed as previously described (19), and the optical density was recorded at 24, 48, 72, and 96h.

### Plasmid construction and dual luciferase reporter assays

Plasmids, including pcDNA3.1-*NEAT1* (pc-*NEAT1*), pcDNA3.1-negative control (pc-NC), and those used for luciferase reporter assays, were designed and synthesized by Riocom Biotech (Shenzhen, China). Cells were transfected using the X-treme Plasmid Transfection Kit (Sigma, St. Louis, MO, USA), and luciferase reporter assays were performed as previously described (20).

### Construction of DCM model rats

Male Sprague-Dawley rats, 6-8 weeks old and weighing 160-180g, were fed a high-fat diet (HFD) consisting of normal powder diet (365 g/kg), lard (310 g/kg), casein (250 g/kg), cholesterol (10 g/kg), vitamins and minerals (60 g/kg), DL-methionine (3 g/kg), yeast powder (1 g/kg), and sodium chloride (1 g/kg), for 2 weeks. Rats were then fasted for 4-6h, and a single dose of STZ (35 mg/kg) dissolved in citrate buffer (pH=4.4) was intraperitoneally administered to induce DM. After 72h, fasting blood glucose levels were determined using a glucometer (Roche, Basel, Switzerland) to confirm the development of DM. Following the successful development of DM, rats were maintained on the HFD until the 20^th^ week. The rats were then divided into the DCM and treatment groups (n=6 rats, each group). Rats in the DCM group were administered 0.9% NaCl by gavage for 4 weeks, while those in the treatment group were administered dendrobium mixture (10 g/kg) and Met (0.18 g/kg) by gavage for 4 weeks.

### Hematoxylin and eosin staining

Heart tissues were collected, fixed in 4% paraformaldehyde, and embedded in paraffin. The embedded tissue samples were sliced into 5 μm thick sections and stained with hematoxylin and eosin (HE) for histological analysis (21).

### Data analysis

Data are presented as the mean ± SEM and were analyzed by one-way ANOVA, followed by Tukey’s multiple comparison test using Prism Software (GraphPad, San Diego, CA, USA). Statistical significance was defined as *p<*0.05.

## RESULTS

### *NEAT1* expression is downregulated in high glucose-treated myocardial cells

**Figure 1:**
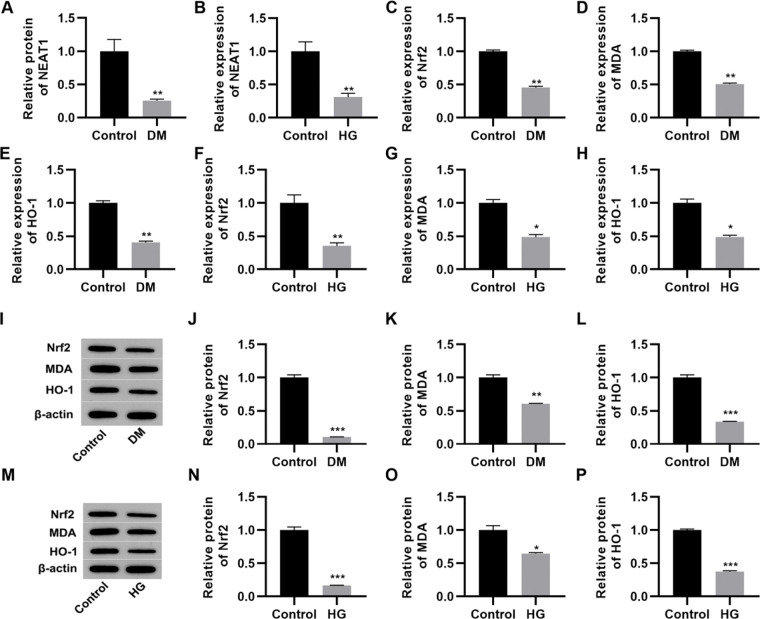
*NEAT1* is downregulated in patients with diabetes mellitus and H9c2 cells maintained in high-glucose medium. A, Expression of *NEAT1* in patients with diabetes mellitus (DM); B, Expression of *NEAT1* in H9c2 cells maintained in high-glucose (HG) medium; C-E, mRNA expression levels of *Nrf2*, *MDA*, and *HO-1* in patients with DM; F-H, mRNA expression levels of *Nrf2*, *MDA*, and *HO-1* in H9c2 cells maintained in HG medium; I-L, Western blotting of Nrf2, MDA, and HO-1 in patients with DM; M-P, Western blotting of Nrf2, MDA, and HO-1 in H9c2 cells maintained in HG medium. *p<0.05, **p<0.01, ***p<0.001, Mann-Whitney U test.

The qRT-PCR results indicated that serum *NEAT1* levels were lower in patients with DM than in their healthy counterparts (Figure 1A, *p*<0.01). To analyze the effect of high glucose on the expression of *NEAT1 in vitro*, we cultured H9c2 cells in medium supplemented with either low (5.5 mmol/L) or high (50 mmol/L) glucose. Cultivation in high glucose resulted reduced *NEAT1* expression compared to its levels in control cells maintained in low glucose (Figure 1B, *p*<0.01). Moreover, we performed qRT-PCR and western blotting to assess the changes in the Nrf2-related pathway and found that Nrf2, MDA, and HO-1 mRNA and protein expression levels were downregulated in both patients with DM and H9c2 cells maintained in high glucose (Figure 1C-P, *p*<0.05, *p*<0.01, *p*<0.001).

### Dendrobium mixture and Met upregulate *NEAT1* expression in H9c2 cells maintained in high glucose

**Figure 2:**
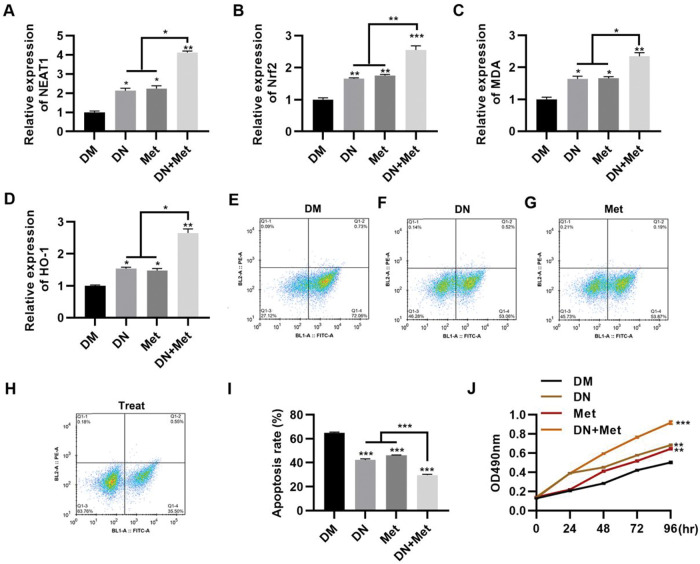
*NEAT1* expression in H9c2 cells maintained in high-glucose medium is upregulated by combination treatment with dendrobium mixture and metformin, which was accompanied by a significant decrease in apoptosis. A-D, The combination of dendrobium mixture (DN) and metformin (Met) promoted the expression of *NEAT1* and Nrf2 pathway-related genes in H9c2 cells maintained in high-glucose (HG) medium. The combination of dendrobium mixture and Met inhibited apoptosis (E-I) and promoted the proliferation (J) of H9c2 cells maintained in HG medium. *p<0.05, **p<0.01, ***p<0.001, Kruskal-Wallis test followed by Dunn’s multiple comparisons test (A-I), Mann-Whitney U test (J).

To evaluate the potential of dendrobium mixture in combination with Met for the treatment of DCM, the effect of dendrobium mixture and Met treatment was assessed in H9c2 cells cultured in HG medium. The combination of dendrobium mixture and Met significantly upregulated the expression levels of *NEAT1* (Figure 2A, *p*<0.05, *p*<0.01) and genes related to the Nrf2 pathway, including *Nrf2*, *MDA*, and *HO-1* (Figure 2B-D, *p*<0.05, *p*<0.01, *p*<0.001). Accumulating evidence suggests that the rate of myocardial cell apoptosis is increased in a HG environment (9,15,22,23). To explore the anti-apoptotic effect of dendrobium mixture and Met on H9c2 cells in a HG environment, we treated cells with dendrobium mixture, Met, and both dendrobium mixture and Met and performed an apoptosis assay with PI and Annexin V to detect apoptosis by flow cytometry. The results showed that while treatment with either dendrobium mixture or Met decreased apoptosis, the combination treatment showed better efficacy (Figure 2E-I, *p*<0.01, *p*<0.001). The results of the MTT assay confirmed that the combination of dendrobium mixture and Met promoted the proliferation of H9c2 cells in HG medium (Figure 2J, *p*<0.01, *p*<0.001).

### Upregulation of *NEAT1* inhibits myocardial cell apoptosis

**Figure 3:**
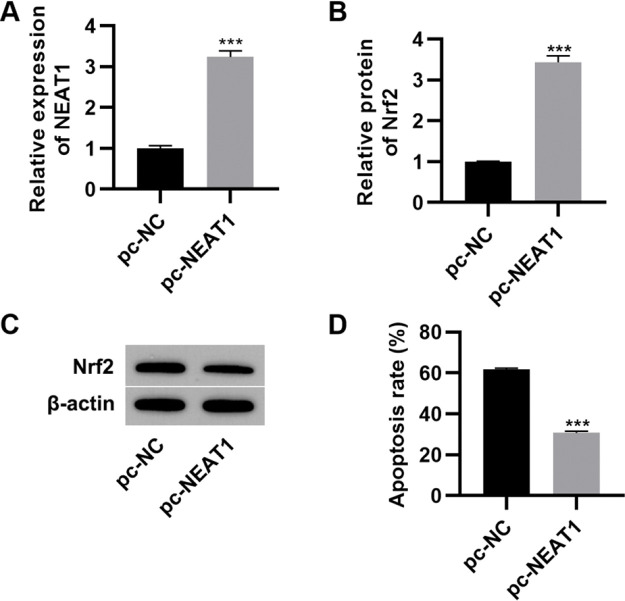
Upregulation of *NEAT1* inhibited the apoptosis of myocardial cells. Transfection of pcDNA3.1-*NEAT1* upregulated the mRNA expression of *NEAT1* (A) and Nrf2 protein levels (B-C) and inhibited apoptosis (D) in H9c2 cells maintained in high-glucose (HG) medium. ***p<0.001, Mann-Whitney U test.

To further investigate the effect of *NEAT1* on myocardial cell apoptosis, we transfected H9c2 cells with a plasmid expressing *NEAT1* (pc-*NEAT1*) or a control plasmid (pc-NC) and cultured them in HG medium. As expected, transfection of pc-*NEAT1* in the myocardial cells significantly upregulated the expression of *NEAT1* (Figure 3A, *p*<0.001), and it also upregulated the expression of Nrf2 (Figure 3B-C, *p*<0.001). Flow cytometry experiments also showed that transfection of pc-*NEAT1* abolished the HG-induced increase in H9c2 cell apoptosis (Figure 3D, *p*<0.001).

### *NEAT1* regulates Nrf2 expression by sponging *miR-23a-3p*

**Figure 4:**
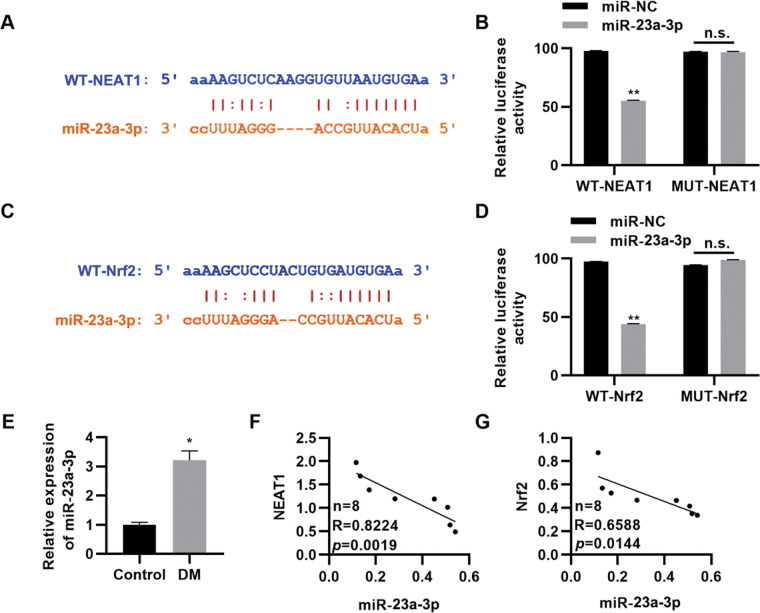
*NEAT1* regulates the expression of Nrf2 by targeting *miR-23a-3p*. A, Identification of a binding site between *NEAT1* and *miR-23a-3p*; B, Dual luciferase reporter assay confirming binding between *NEAT1* and *miR-23a-3p*; C, Identification of a binding site between *Nrf2* and *miR-23a-3p*; D, Dual luciferase reporter assay confirming binding between *Nrf2* and *miR-23a-3p*; E, The expression of *miR-23a-3p* is upregulated in patients with diabetes mellitus (DM); F-G, The expression levels of *miR-23a-3p* are negatively correlated with the levels of *NEAT1* and *Nrf2*. *p<0.05, **p<0.01, Mann-Whitney U test.

To further clarify the molecular mechanism by which *NEAT1* regulates Nrf2 expression, we predicted the regulatory targets of *NEAT1* using StarBase (http://starbase.sysu.edu.cn/index.php). We noted that *NEAT1* had a potential binding site in *miR-23a-3p* (Figure 4A), which was validated by a dual luciferase reporter assay in H9c2 cells (Figure 4B, *p*<0.01). Our findings also suggested potential binding between *miR-23a-3p* and *Nrf2* (Figure 4C-D, *p*<0.01). Subsequently, we measured the expression levels of *miR-23a-3p* in patients with DM and found that *miR-23a-3p* expression levels were upregulated in these patients (Figure 4E, *p*<0.05). Spearman’s correlation analysis also showed negative correlations between *NEAT1* and *miR-23a-3p* levels and between *miR-23a-3p* and Nrf2 levels in patients with DM (Figure 4G).

### Expression levels of *NEAT1* and Nrf2 are altered in rats with STZ-induced DCM

**Figure 5:**
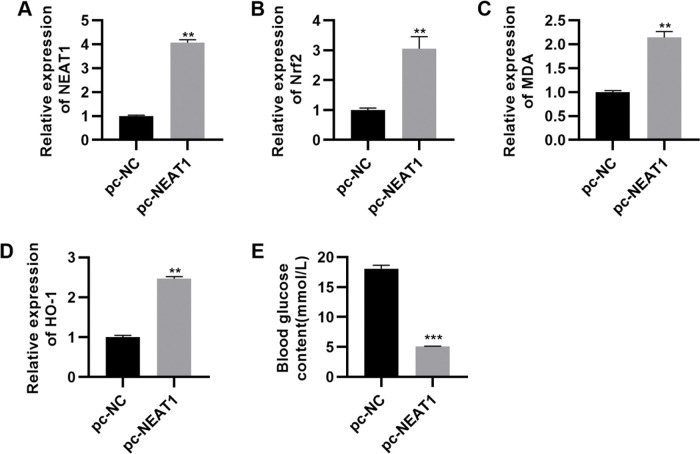
pc-NEAT1 upregulated the expression of *NEAT1* and Nrf2 pathway-related genes. A-D, Transfection of pcDNA3.1-*NEAT1* upregulated the expression of *NEAT1* and Nrf2 pathway-related genes and E, decreased blood glucose levels in rats with diabetic cardiomyopathy (DCM). **p<0.01, ***p<0.001, Mann-Whitney U test.

To confirm the significance of *NEAT1* in DCM, we upregulated the expression of *NEAT1* by transfecting a NEAT1 expression plasmid (pc-*NEAT1*) in rats with DCM. *NEAT1* was downregulated in rats with STZ-induced DM, while Nrf2 was upregulated in these rats (Figure 5A-D, *p*<0.01). In rats transfected with pc-*NEAT1*, Nrf2 was downregulated (Figure 5A-D, *p*<0.01), which was accompanied by a sharp decrease in blood glucose levels (Figure 5E, *p*<0.001).

### Treatment with dendrobium mixture and Met improves cardiac morphology in rats with STZ-induced DM

**Figure 6:**
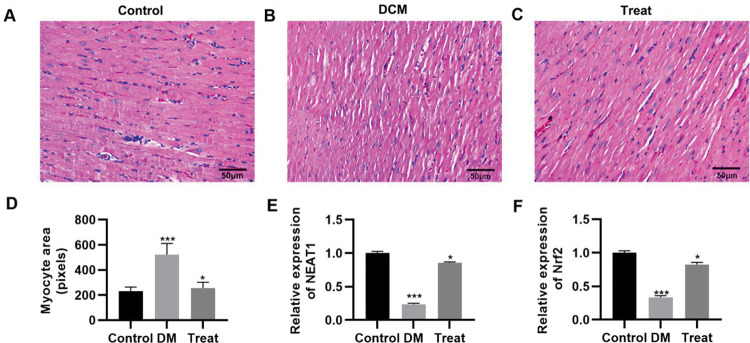
Treatment with the combination of dendrobium mixture and metformin improved the morphology of heart tissues in rats with streptozotocin-induced diabetes mellitus. A-C, Hematoxylin and eosin staining of the heart tissue of the rats in each group; D, Treatment with dendrobium mixture and metformin improved myocardial cell size in streptozotocin-induced diabetic rats; and E-F, upregulated the expression of *NEAT1* and *Nrf2*. *p<0.05, ***p<0.001, Kruskal-Wallis test followed by Dunn’s multiple comparisons test. DCM, diabetic cardiomyopathy.

To validate the *in vivo* efficacy of the combination of dendrobium mixture and Met, we constructed a rat model of DCM. The DCM model rats were then treated with the combination of dendrobium mixture and Met, and the pathological changes in cardiac tissues were assessed. As indicated by HE staining, rats with DCM had cardiac hypertrophy, and their myocardial cells showed an abnormal morphology, with enlarged nuclei, widened intercellular spaces, and a reduced number of longitudinal connections. These abnormalities were reversed in rats that received the combination treatment (Figure 6A-D). The qRT-PCR results also indicated that the combination of dendrobium mixture and Met upregulated the expression levels of *NEAT1* and *Nrf2* (Figure 6E-F, *p*<0.05, *p*<0.001).

## DISCUSSION

The findings of this study suggest that the combination of dendrobium mixture and Met had a protective effect against DCM as simulated by either HG levels in cells or STZ administration in rats. This treatment combination decreased apoptosis of H9c2 cells cultured in HG medium.

**Figure 7:**
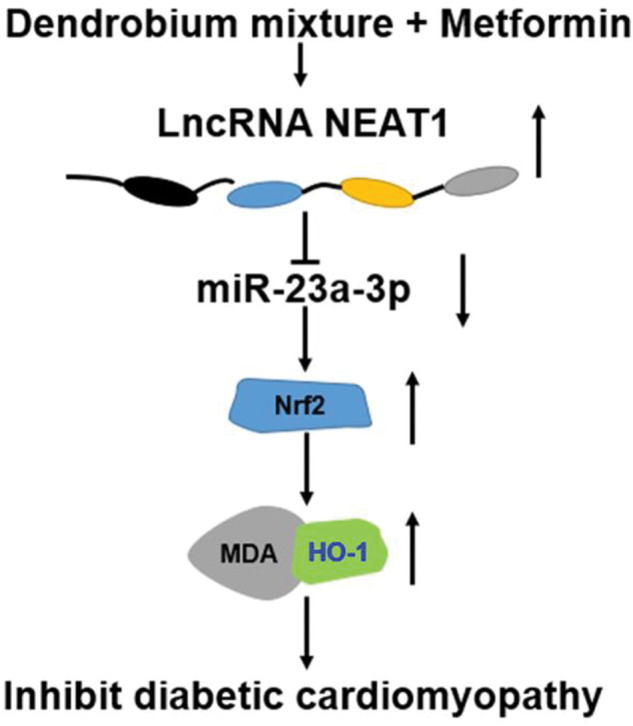
Proposed mechanism of action for the combination of dendrobium mixture and metformin in the treatment of diabetic cardiomyopathy. *NEAT1*, acting as a sponge of *miR-23a-3p*, can regulate the expression of MDA and HO-1 by targeting *Nrf2*.

We also determined the effect of the combination of dendrobium mixture and Met on the expression of *NEAT1* in both myocardial cells and rats with DCM. Various lncRNAs have been shown to play key roles in the pathogenesis of a numerous diseases, including cardiovascular diseases and DM as well as their related complications (24-26) was significantly downregulated in patients with DM, HG-treated myocardial cells, and heart tissues from model rats with DCM. However, this downregulation was reversed by combined treatment with dendrobium mixture and Met. Based on the observed interactions among *NEAT1*, *miR-23a-3p*, and *Nrf2*, we inferred that dendrobium mixture and Met may reduce apoptosis of myocardial cells and improve the function and morphology of heart tissues in rats with DCM by upregulating the expression of *NEAT1* through *miR-23a-3p* and *Nrf2*. The proposed mechanism for the role of lncRNA *NEAT1* in DCM is shown in Figure 7.

The main function of Nrf2 is to protect cells from oxidative stress-induced damage by modulating antioxidant enzyme expression (27,28). This function is mediated via the Nrf2/antioxidant response element pathway, which regulates the activity of various enzymes, including the antioxidant enzymes MDA and HO-1 (29-31). However, there is no evidence suggesting that dendrobium mixture can activate the Nrf2/ARE pathway to protect cells from DCM-induced damage.

The characteristic features of DCM are the deterioration of myocardial function in diastole and myocardial cell apoptosis and fibrosis. In a HG environment, myocardial cells are more susceptible to ROS-induced damage. As ROS activate apoptosis- and fibrosis-related pathways (7), this is one of the causes of DCM (5-7,32). More importantly, oxidative and nitrative stress in DCM have been shown to be closely associated with inflammation, which, in turn, may promote the progression of myocardial fibrosis (33). The finding that dendrobium mixture in combination with Met reduced the apoptosis of myocardial cells in the hearts of patients with DM and was accompanied by upregulation of Nrf2, shows the significant impact of this treatment against oxidative and/or nitrative stress-induced damage and its protective effect in myocardial cells.

In conclusion, the results of this study indicated that the combination of dendrobium mixture and Met promoted the expression of the lncRNA *NEAT1* in DCM, which reduced the expression of *miR-23a-3p* and upregulated the expression of *Nrf2* to inhibit the apoptosis of myocardial cells. Thus, dendrobium mixture and Met may be an efficient treatment strategy for DCM.

## AUTHOR CONTRIBUTIONS

Qin W, Zhao X, and Yu S conceived the project and designed and performed the experiments. Tai J and Qin G analyzed the data. Yu S wrote and revised the manuscript
